# Synthesis and Characterization of Anatase TiO_2_ Microspheres Self-Assembled by Ultrathin Nanosheets

**DOI:** 10.3390/ma14112870

**Published:** 2021-05-27

**Authors:** Jian Di, Haibo Yan, Zhuoyu Liu, Xing Ding

**Affiliations:** 1 State Key Laboratory of Isotope Geochemistry, Guangzhou Institute of Geochemistry, Guangzhou 510640, China; dijian@gig.ac.cn (J.D.); lzhy1598@163.com (Z.L.); 2 CAS Center for Excellence in Deep Earth Science, Guangzhou 510640, China; 3 College of Earth and Planetary Sciences, University of Chinese Academy of Sciences, Beijing 100049, China; yanhaibo@gig.ac.cn

**Keywords:** anatase, titanium dioxide, self-assembled microspheres, potassium fluorotitanate, high (001) facets

## Abstract

In this paper, we report a novel and simple method for synthesizing the microspheres self-assembled from ultrathin anatase TiO_2_ nanosheets with a high percentage of (001) facets via the hydrolysis process of the single-reagent (potassium fluorotitanate). We then used optical microscopy, scanning electron microscopy, and high-resolution confocal laser Raman spectroscopy to characterize the microspheres generated under different conditions. The study found that the size of the anatase TiO_2_ microspheres synthesized was 0.5–3 μm. As the synthesis time increased, the corroded surface of the microspheres gradually increased, resulting in the gradual disappearance of the edges and corners of the anatase nanosheets. The exposure percentage of the (001) facets of ultrathin anatase nanosheets synthesized for 2 h at 180–200 °C are close to 100%. The microsphere whose surface is completely covered by these anatase nanosheets also has nearly 100% exposed (001) facets. This new anatase nanosheet-based self-assembled microsphere will have great application potential in pollution prevention, environmental protection, and energy fields.

## 1. Introduction

Nanocrystalline titanium dioxide (TiO_2_) has good chemical and physical stability and excellent photocatalytic performance [1,2,3]. Due to its scientific and technological importance, it is widely used in optoelectronic devices, sensors, solar cells, and photonic crystals [4,5].

There are three nano-TiO_2_ polymorphs reported, i.e., anatase (AT), rutile (RT), and brookite (BT) [1], in which the anatase-type TiO_2_ shows the best photocatalysis and photoelectric performance [5]. Different synthesis methods can produce various anatase nano-TiO_2_ crystals with distinct sizes and morphology, resulting in discrepant physicochemical properties and photocatalytic performance. In the previous studies, anatase nano-TiO_2_ truncated octahedron, rod, wire, tube, belt, sheet, film, and sphere structures were synthesized [1,6].

Recently, a three-dimensional spherical structure assembled by anatase nanosheets with a high percentage of (001) facets has been given wide attention. The exposure rate of the (001) facets in this kind of structure is very high, reaching nearly 100% [7]. For anatase nano-TiO_2_, theoretical and experimental studies have demonstrated that the (001) facets are highly reactive towards photocatalytic performance [6]. Therefore, the anatase nano-TiO_2_ spheres usually have a higher photocatalytic capability than other nanostructures and thus can be widely used in pollution prevention and environmental protection [8,9,10,11,12]. For instance, a flower-like TiO_2_ nanosphere structure with highly exposed (001) facets was synthesized through a hydrothermal reaction of titanium powder, hydrofluoric acid, and water at 120 °C, which has two times stronger reducing pollution ability than normal nano-TiO_2_ [8]. Besides, more recent studies have also shown that this type of microsphere assembled from anatase nanosheets with a high percentage of (001) facets displays excellent photoelectric properties, causing a great application potential in energy fields, including solar cells and lithium batteries [9,13,14,15,16]. As an example, the synthesized anatase TiO_2_ nanosheets-based hierarchical spheres with over 90% (001) facets were used as photoanodes of dye-sensitized solar cells, demonstrating that it can generate an energy conversion efficiency of 7.51% [13].

Here, a novel and simple synthesized method for preparing anatase TiO_2_ microspheres self-assembled by ultrathin nanosheets was reported, which displayed nearly 100% exposed (001) facets. During the synthesis, only a single reagent, potassium fluorotitanate, was used as the starting material, while an easy-to-use hydrothermal procedure was adopted. This kind of anatase TiO_2_ microsphere self-assembled by ultrathin nanosheets most likely has great application potential in reducing pollution, environmental protection, and energy fields.

## 2. Experimental Section

### 2.1. Instruments and Reagents

**Figure 1:**
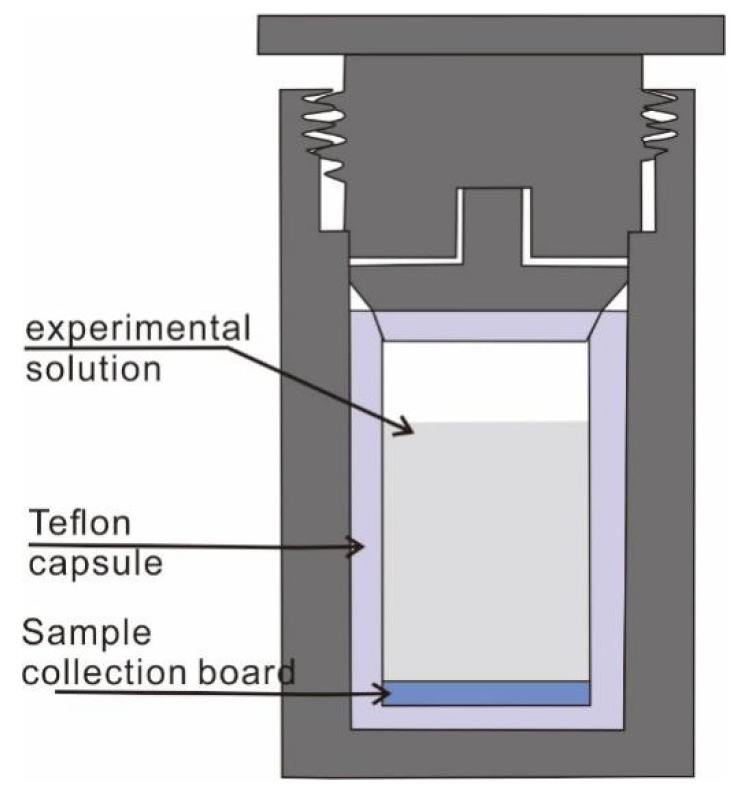
Schematic diagram of a stainless steel hydrothermal reactor with Teflon liner.

The instruments for sample synthesis include the hydrothermal reactors consisting of a stainless steel bushing, polytetrafluoroethylene (Teflon) liner (Figure 1), a muffle furnace (±0.2 °C, FO510C, YAMATO, Osaka, Japan), and a constant-temperature drying oven (±1 °C, DGG-9070B, Jiangdong, Guangzhou, China).

A SUPRA 55 SAPPHIRE field-emission scanning electron microscope (SEM)(Carl Zeiss, Oberkochen, Germany) equipped with an Oxford Inca250 X-Max20 energy dispersive spectrometer (Oxford, UK) was used for morphological analysis of the synthesized crystals at South China University of Technology, China. Qualitative and structure analysis of the synthesized crystals were conducted by a WITec alpha300R high-resolution confocal Raman spectrometer (WITec, Ulm, Germany), Germany, which is equipped with three lasers (488, 532, and 633 nm), three gratings (300, 600, and 1800 grooves/mm), a back-illuminated charge-coupled detector (1600 × 200 pixels), and a Carl Zeiss Microscope (Carl Zeiss, Oberkochen, Germany) at the State Key Laboratory of Isotope Geochemistry, Guangzhou Institute of Geochemistry, China [17].

The starting reagent, potassium fluorotitanate (Figure S1)(K_2_TiF_6_, AR 99.5%, Aladdin, Shanghai, China), is the most stable chemical among Ti-F complexes at room temperature and hydrolyzes at high temperature and high pressure. Its dilute solutions of 0.02–0.04 mol/L contain impurities (Si, Al, Na, Zn, etc.) no more than 3.7 μg/mL (Figure S2) [18]. Note that potassium fluorotitanate is a high toxicity chemical, and someone must protect himself from poisoning by wearing a protective mask, clothing, and gloves when working with it. All the operations related to the starting and resultant solutions must be made in the fume hood.

### 2.2. Synthesis Method

In this study, self-assembled microspheres were synthesized by an on-site precipitation hydrothermal method or a drop-casting deposition method. Potassium fluorotitanate (K_2_TiF_6_) at room temperature was used to prepare a dilute solution at a concentration of 0.01–0.04 mol/L by adding ultrapure water (Figures S3 and S4). The dilute potassium fluorotitanate solution was then added into the hydrothermal autoclave lined with polytetrafluoroethylene (PTFE), accounting for 20–90% volume of the hydrothermal reactor.

In an on-site precipitation hydrothermal method, a thin sheet was placed at the bottom of the hydrothermal reactor. As a collection board, the thin sheet is made of polytetrafluoroethylene or inert metal, such as gold, with a smaller diameter than the inner hole of the hydrothermal reactor. The hydrothermal reactor was tightened and sealed and then heated to 160–200 °C in the FO510C muffle furnace (YAMATO, Osaka, Japan) for 1–5 h. After that, the reactor was cooled entirely to room temperature and opened in the fume hood. The collection board was taken out and soaked in deionized water two to three times and then dried in an oven at 60 °C. As a comparison, in a drop-casting deposition method, the potassium fluorotitanate dilute solution was added directly into the hydrothermal reactor. Then the solution was heated to 160–200 °C and extracted. Finally, the extracted solution was dropped on the surface of a copper or glass sheet. The sheet was then dried in an oven at 60 °C. All of the resulting products involved in the microspheres self-assembled from anatase ultrathin nanosheets with a high percentage of (001) facets.

## 3. Experimental Results and Discussion

During the synthesis, the dissolved starting material, K_2_TiF_6_, became unstable with increasing temperature and vapor pressure due to Ti(IV) cation’s hydrolysis [18,19,20]. The cumulative hydrolysis reaction and formation of titanium oxide precipitates (Figure S5) can be described as [18,19,21]:

(1) $${\mathrm{TiF}}_{6}^{2-}+2H_{2}O\Leftrightarrow {\mathrm{TiO}}_{2}↓+4\mathrm{HF}+2F^{-}$$

The above reaction shows a strong dependence on temperature and initial solution concentration. Previous studies demonstrated that the hydrolysis rate of 0.02 mol/L K_2_TiF_6_ solution is 61.5% at 200 °C and 78.5% at 250 °C, respectively, while that of 0.04 mol/L K_2_TiF_6_ solution is 27.1% at 200 °C and 50.0% at 250 °C, respectively [18]. Therefore, in this study the hydrolysis rate of K_2_TiF_6_ solution is no more than 61.5%. Representative synthesis processes, conditions, and results are shown in Table 1. All precipitate samples have a microsphere structure with a size of 0.5–3 μm. Due to the divergence of anatase nanosheets synthesized, e.g., the percentage of (001) facets and thickness of anatase nanosheets, the microspheres display discrepant appearance.

### 3.1. Raman Analysis of Experimental Products

**Figure 2:**
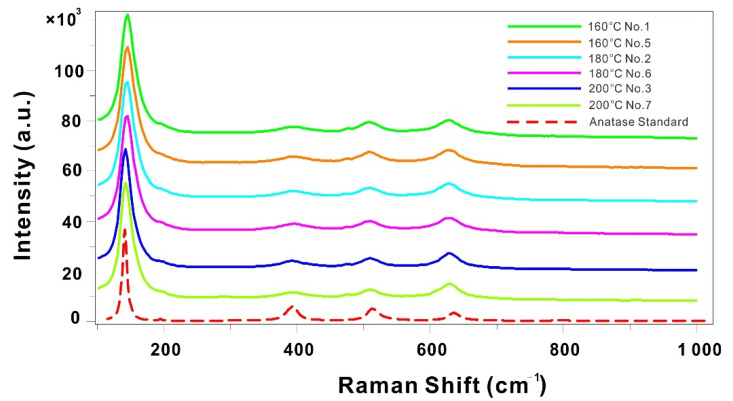
Representative Raman spectra of self-assembled microspheres synthesized on various conditions. The standard Raman spectrum of anatase in Asker, Norway, is selected from the RRUFF database [24].

Figure 2 shows the representative Raman spectra of all microsphere samples as well as a standard anatase sample, all of which coincide in the peak positions. It suggests that all the products synthesized at various temperatures are anatase-type TiO_2_ (Figure S6). This is consistent with previous studies [22,23], which demonstrated that anatase-type TiO_2_ is stable below 400 °C and at low pressures (<10 kbars). Compared with that of the standard anatase sample, the peak intensities of the synthesized microspheres at 390 cm^−1^ obviously decrease, whereas their peak widths become larger. This is most likely due to the increase in porosity leading to changes in the properties of the microspheres.

### 3.2. Temperature Dependence on the Microspheres’ Morphology

**Figure 3:**
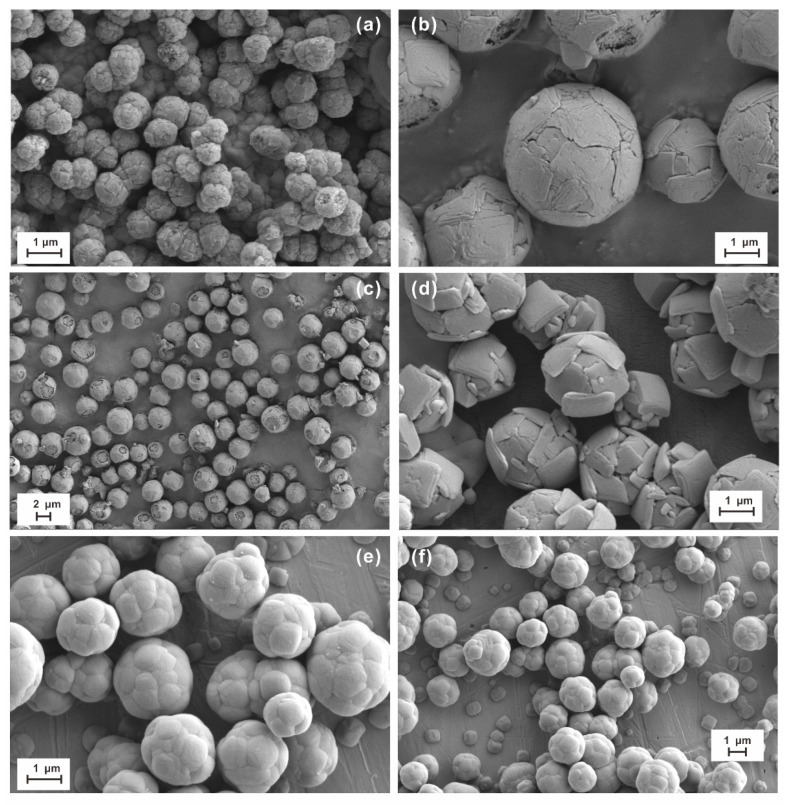
Representative SEM micrographs of microsphere samples synthesized by an on-site precipitation hydrothermal method: No. 1 for 3 h at 160 °C (**a**); No. 2 for 2 h at 180 °C (**b**,**c**); No.3 for 2 h at 200 °C (**d**); and No. 4 for 5 h at 200 °C (**e**,**f**).

We analyzed the micro-morphology of the microspheres that were self-assembled from anatase ultrathin nanosheets at different temperatures—our goal was to determine the difference in the morphology of the microspheres synthesized at different temperatures. The results indeed show the temperature dependence on the microsphere’s morphology. In the on-site precipitation hydrothermal method (Figure 3), the anatase nanosheets forming the microspheres with highly exposed (001) facets synthesized for 2 h at 200 °C (Figure 3d) display more euhedral and are thicker than those at 160 °C (Figure 3a) and 180 °C (Figure 3b,c).

The microsphere in 160 °C (Figure 3a) is composed of euhedral and subhedral high (001) anatase ultrathin nanosheets with a size of 1–3 μm. Anatase ultrathin nanosheets with ~100% high (001) facets adhered to each other and cover the whole sphere surface, resulting in the smooth surface and almost 100% exposed (001) facets of the microspheres. Some microspheres show a loose internal structure. Compared with the microspheres (160 °C, 3 h), the particle sizes are 1–2 μm in 180 °C for 2 h and 200 °C for 2 h. The surface of the microspheres (180 °C, 2 h) is not smooth. It is evident that the crystal shape was poor and largely eroded in 200 °C for 2 h.

**Figure 4:**
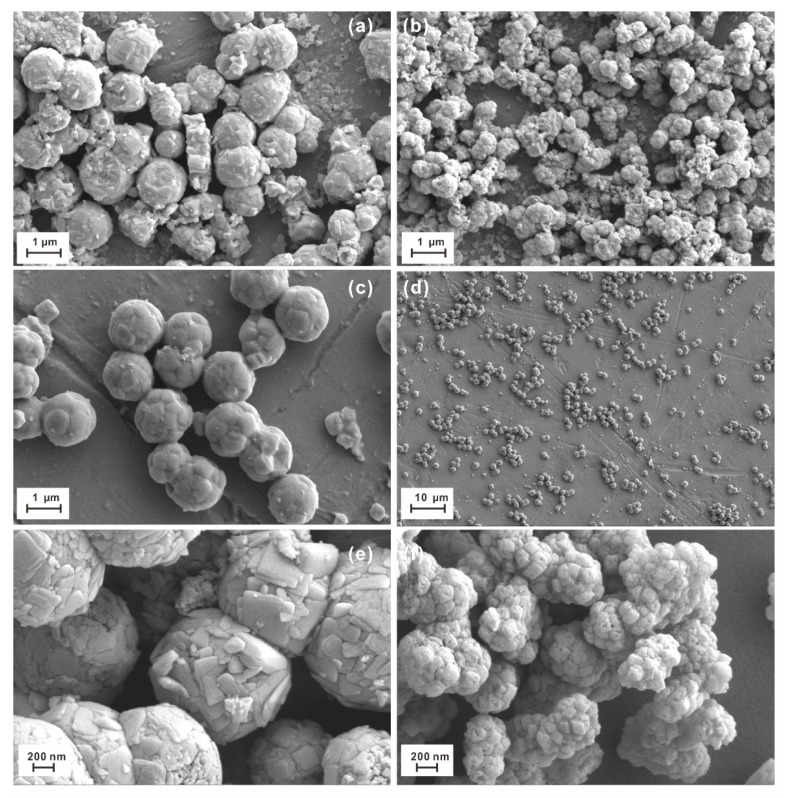
Representative SEM micrographs of microsphere samples synthesized by a drop-casting deposition method: No. 5 for 2 h at 160 °C (**a**); No. 6 for 5 h at 180 °C (**b**); No.7 for 5 h at 200 °C (**c**,**d**); No.8 for 2 h at 200 °C (**e**); and No. 9 for 3 h at 160 °C (**f**).

Similarly, in the drop-casting deposition method, the anatase nanosheets synthesized for 2 h at 200 °C (Figure 4e) show more euhedral than those synthesized at 160 °C, not only for 2 h (Figure 4a) but for 3 h (Figure 4f). The temperature dependence on the anatase’s morphology is consistent with previous studies [1].

The particle size of microspheres produced under different synthesis conditions is 0.8–1 μm (200 °C, 2 h), 0.5–1.5 μm (200 °C, 2 h), and 0.5–1 μm (200 °C, 3 h), respectively.

Ding et al. found that the anatase’s surface structure changes from the nanosheet with a very high percentage of (001) facets at <200 °C to the truncated octahedron with a very low percentage of (001) at 300 °C, suggesting the temperature effect on the exposed percentage of (001) facets for the anatase [22]. In this study, the surface of the microspheres synthesized at 180 °C and especially at 200 °C are almost completely covered by the ultrathin anatase nanosheets with exposed (001) facets of close to 100%. This causes the exposed (001) facets of the self-assembled microspheres to be close to 100%.

### 3.3. The Effect of Synthesis Time on the Microspheres’ Morphology

The difference in the morphology of microspheres undergoing different synthesis times at similar temperatures also indicates the time effect. As shown in Figure 3, the ultrathin TiO_2_ nanosheets forming the microspheres synthesized by the on-site precipitation hydrothermal method at 200 °C for 2 h display complete shapes with distinct edges and corners (Figure 3d); by contrast, those nanosheets synthesized at 200 °C for 5 h were damaged and lost most of their edges and corners, making better roundness for the microspheres (Figure 3e,f); the size of the nanosheets synthesized was about 1–2 μm.

Likewise, in the drop-casting deposition method, the synthesized anatase TiO_2_ nanosheets in the microspheres at 200 °C for 2 h show euhedral and subhedral morphology (Figure 4e), whereas those at 200 °C for 5 h exhibit low crystallinity (Figure 4c,d). The particle size of the microspheres formed at 200 °C for 5 h is 1–2 μm. Also, the anatase was largely eroded.

The microsphere at 180 °C (Figure 4b) was composed of xenomorphic and subhedral high (001) anatase nanosheets with a size of 0.5–1 μm; most of the anatase nanosheets failed to form the self-assembled microsphere.

Although fluorine can act as a morphology-controlling agent to stabilize the growth of the (001) facets of anatase [6], this study suggests that it can also corrode and damage the anatase shape, as previously reported [22,25]. Therefore, controlling the synthesis time is a key factor in synthesizing the anatase nanosheets and microspheres with excellent shapes and reactivity.

### 3.4. Comparison of the Synthesis Methods

Figure 3 and Figure 4 show the representative SEM micrographs of self-assembled microspheres synthesized by the on-site precipitation hydrothermal method and the drop-casting deposition method, respectively. They clearly display that the anatase nanosheets and microspheres synthesized by the on-site precipitation hydrothermal method have better crystallinity, shapes, and dimensions than those synthesized by the drop-casting deposition method. As mentioned above, the self-assembled microspheres and therein anatase nanosheets have significant temperature dependence. In the on-site precipitation hydrothermal method, the potassium fluorotitanate can be hydrolyzed under constant-temperature conditions, which ensures the constant growth of anatase nano-TiO_2_ [22]. By contrast, in the drop-casting deposition method, the potassium fluorotitanate hydrolyzes first at high temperatures to produce the first-generation anatase nano-TiO_2_ and continues to hydrolyze at low temperatures during the drying process to form the second anatase nano-TiO_2_, leading to the formation of many small, xenomorphic, and fragmentary anatase nanocrystals (Figure 4). Although both the methods can synthesize the anatase nanosheet-based microspheres with the high exposed (001) facets, it is evident that the microspheres synthesized by the on-site precipitation hydrothermal method have a more stable structure and thus better physicochemical properties as well as photocatalytic and photoelectric performance [1,3]. In addition, even if the materials for the collection board are different, it seems to have an insignificant influence on the anatase and microsphere’s morphology compared to the temperature, synthesis time, and synthesis method under the same conditions.

## 4. Conclusions

The three-dimensional spherical structure self-assembled by ultrathin anatase nanosheets with nearly 100% exposed (001) facets was synthesized in this study. We compared two methods, i.e., the on-site precipitation hydrothermal method and the drop-casting deposition method, using a single reagent (potassium fluorotitanate) as the starting material. The results show that the microspheres synthesized by the on-site precipitation hydrothermal method have better crystallinity, shapes, dimensions, and great application potential in photocatalytic and photoelectric fields. Besides, the results demonstrated that the temperature and synthesis time had significant effects on the morphology of microspheres and therein anatase nanosheets. An increase in the synthesis time could lead to corrosion on the surface of the microsphere because of the fluorine’s role in the hydrothermal solutions. As a result, the anatase nanosheet-based microspheres synthesized at 180–200 °C and for 2 h show the best appearance.

## Figures and Tables

**Table 1:** Preparation of anatase nano-TiO_2_ microspheres under different conditions *.

Experiment Number	Concentration of the Solution	Collection Board	Temperature	Synthesis Time	Particle Size
1	0.04 mol/L K_2_TiF_6_ dilute solution	PTFE sheet	160 °C	3 h	1–3 μm
2	0.04 mol/L K_2_TiF_6_ dilute solution	PTFE sheet	180 °C	2 h	1–2 μm
3	0.02 mol/L K_2_TiF_6_ dilute solution	PTFE sheet	200 °C	2 h	1–2 μm
4	0.04 mol/L K_2_TiF_6_ dilute solution	Gold flake	200 °C	5 h	1–2 μm
5	0.04 mol/L K_2_TiF_6_ dilute solution	Copper flake	160 °C	2 h	0.5–1.5 μm
6	0.02 mol/L K_2_TiF_6_ dilute solution	Copper flake	180 °C	5 h	0.5–1 μm
7	0.04 mol/L K_2_TiF_6_ dilute solution	Copper flake	200 °C	5 h	1–2 μm
8	0.04 mol/L K_2_TiF_6_ dilute solution	Glass sheet	200 °C	2 h	0.8–1 μm
9	0.04 mol/L K_2_TiF_6_ dilute solution	Glass sheet	160 °C	3 h	0.5–1 μm

* Experiment No. 1–4 are conducted by the on-site precipitation hydrothermal method, while the No. 5–9 by the drop-casting deposition method.

## Data Availability

Data sharing is not applicable to this article.

## Supplementary Materials

The following are available online at https://www.mdpi.com/article/10.3390/ma14112870/s1, Figure S1: Micrograph of potassium fluorotitanate powder (upper) and its crystal Structure (lower).; Figure S2: XRD patterns of the potassium fluorotitanate powder.; Figure S3: Raman spectra of the potassium fluorotitanate crystal (upper) and solution (lower).; Figure S4: ATR-IR (a), FTIR (b), and Raman (c) spectra of the potassium fluorotitanate (the data are from the Aladdin website). Figure S5: EDX analysis of the anatase TiO_2_ microsphere.; Figure S6: Raman spectra of the anatase TiO_2_ microsphere.
