# Supplementary material for: Synthesis and Characterization of Anatase TiO2 Microspheres Self-Assembled by Ultrathin Nanosheets
Source: Materials (Basel). 2021 May 27;14(11):2870. doi: 10.3390/ma14112870 (PMC8198224; doi:10.3390/ma14112870)
Supplement: Supplementary file 1 [file materials-14-02870-s001.zip › materials-1216921-supplementary.pdf]

materials

Supplementary Material

Synthesis and Characterization of Anatase TiO_2 Microspheres Self-Assembled by Ultrathin Nanosheets

Jian Di [1,2,3], Haibo Yan [3], Zhuoyu Liu [1] and Xing Ding [1,2,3] *

[1] State Key Laboratory of Isotope Geochemistry, Guangzhou Institute of Geochemistry, Guangzhou 510640, China;
dijian@gig.ac.cn (J.D.); lzhy1598@163.com (Z.L.)
[2] CAS Center for Excellence in Deep Earth Science, Guangzhou 510640, China
[3] College of Earth and Planetary Sciences, University of Chinese Academy of Sciences, Beijing 100049, China; yanhaibo@gig.ac.cn
* Correspondence: xding@gig.ac.cn

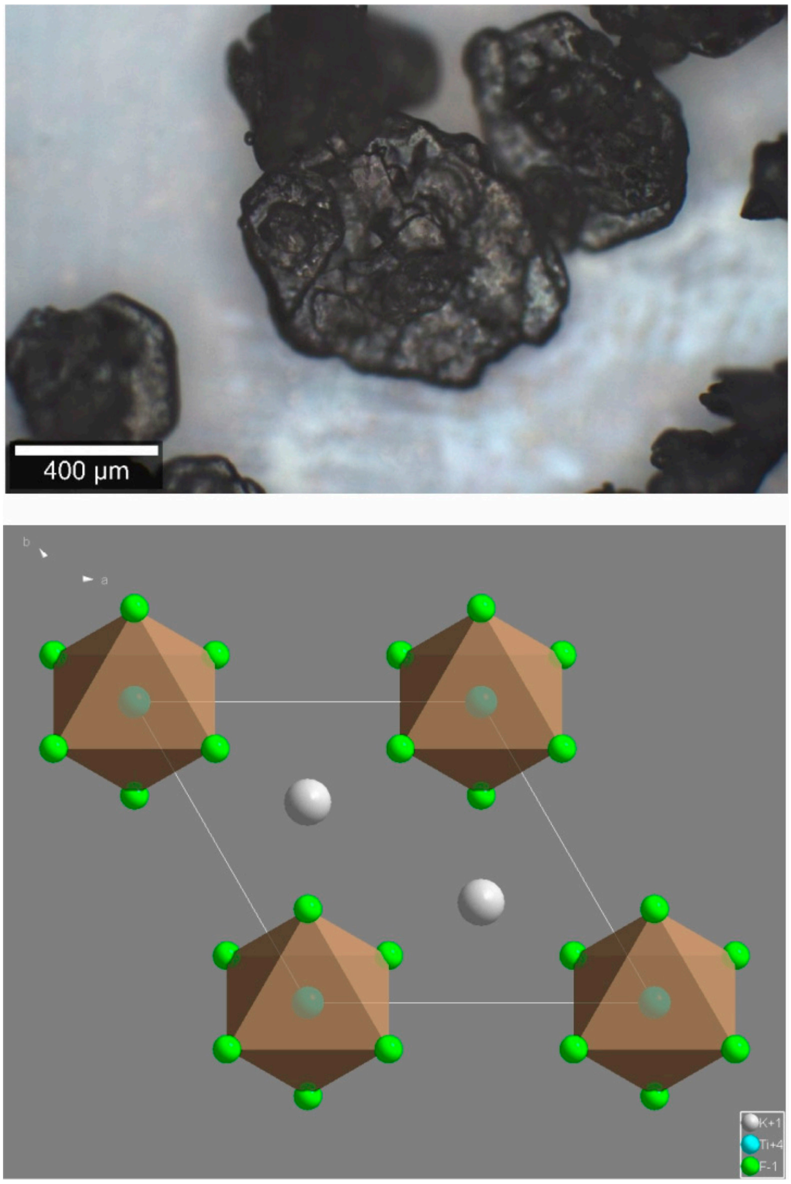

Figure S1. Micrograph of potassium fluorotitanate powder (upper) and its crystal Structure (lower).

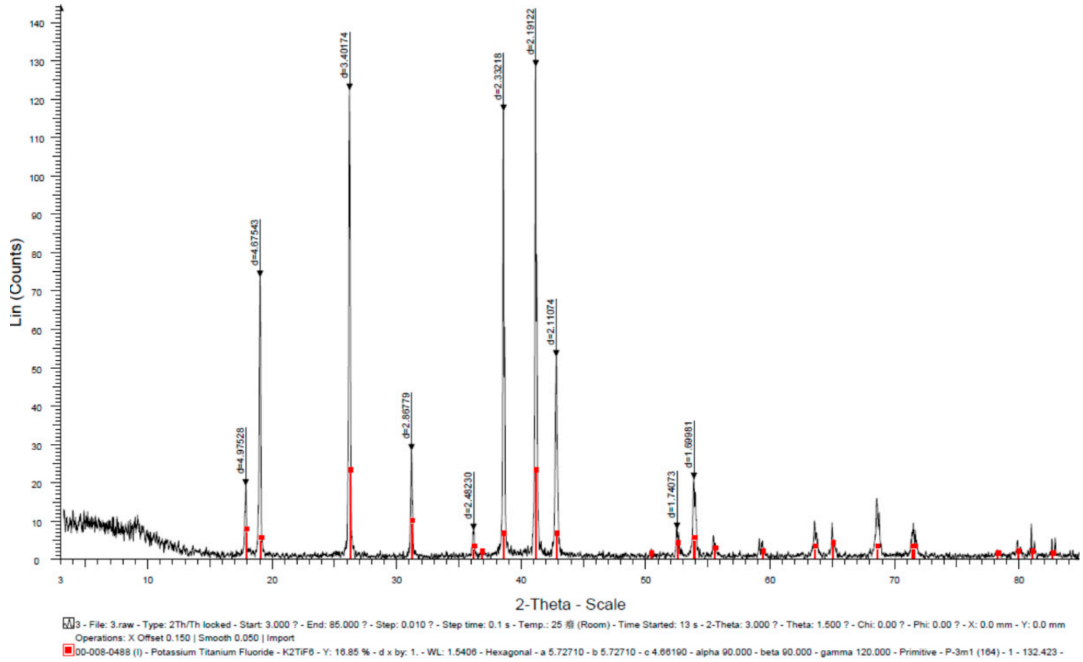

Figure S2. XRD patterns of the potassium fluorotitanate powder.

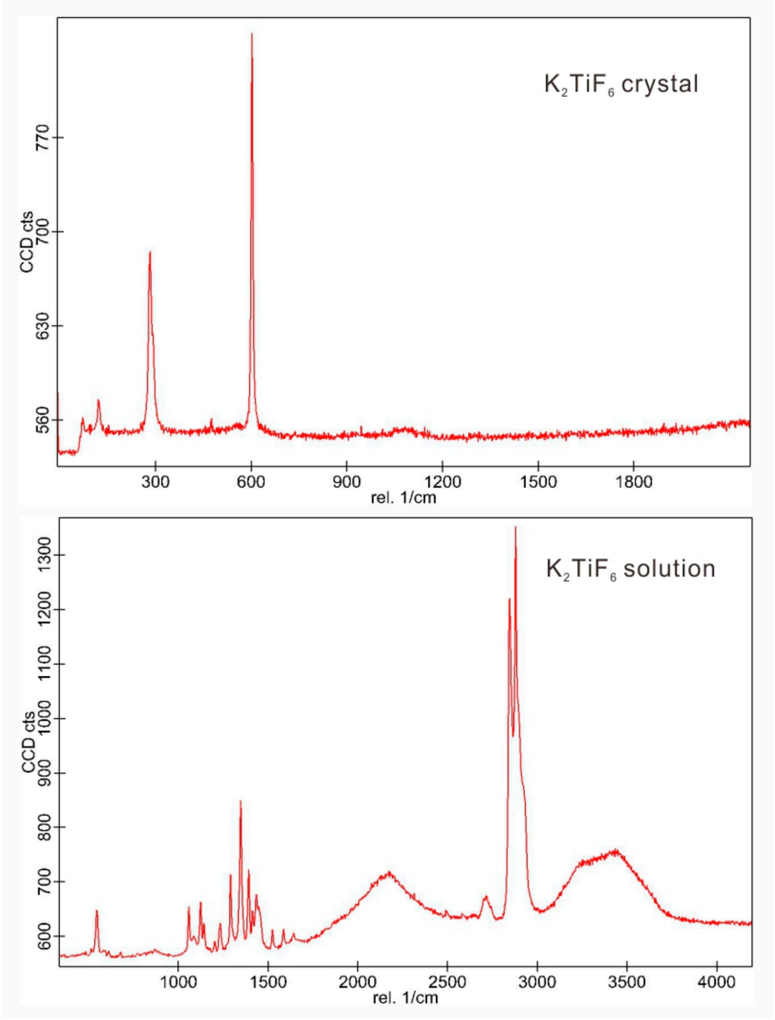

Figure S3. Raman spectra of the potassium fluorotitanate crystal (upper) and solution (lower).

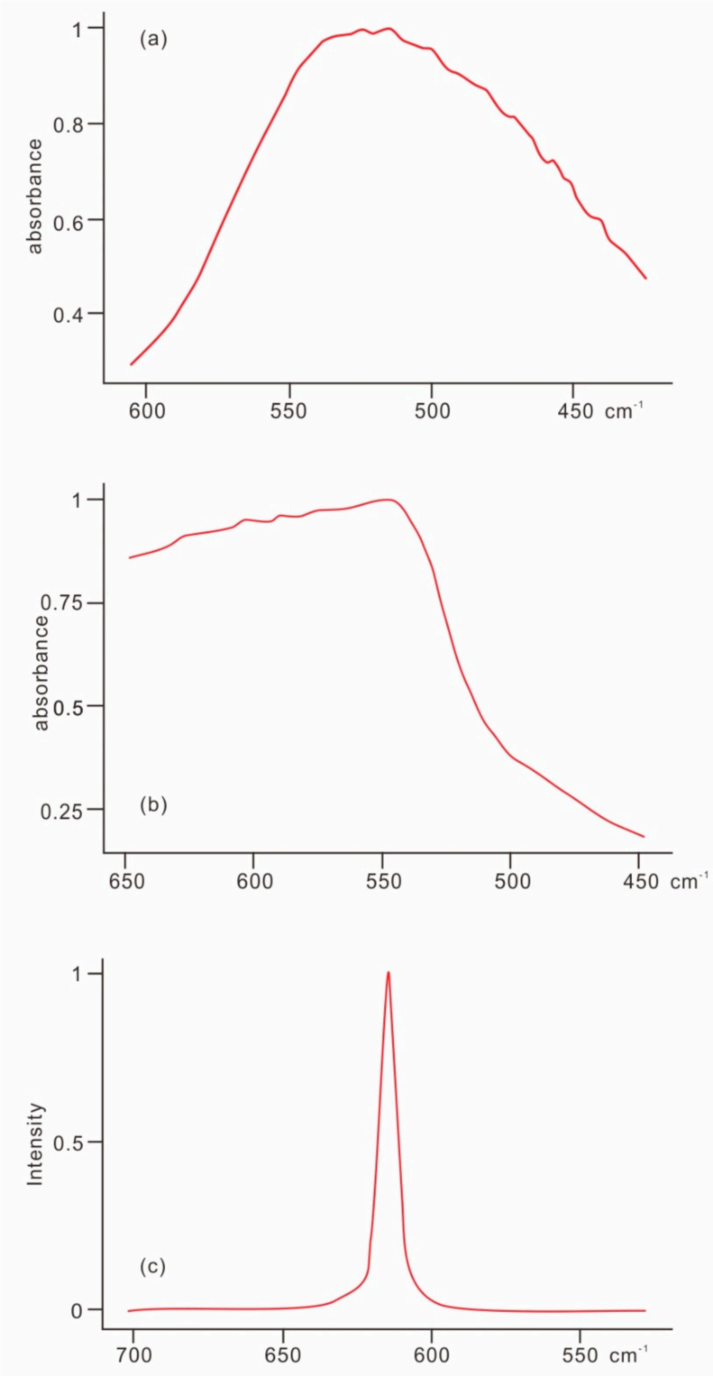

Figure S4. ATR-IR (a), FTIR (b), and Raman (c) spectra of the potassium fluorotitanate (the data are from the aladdin website).

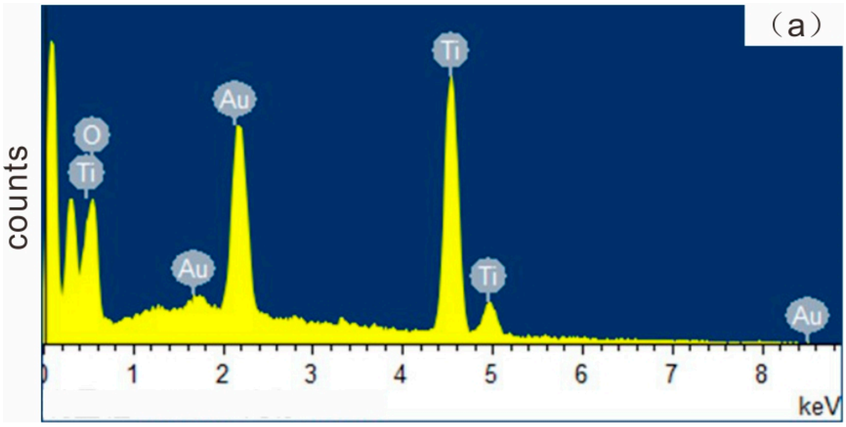

Figure S5. EDX analysis of the anatase TiO_2 microsphere.

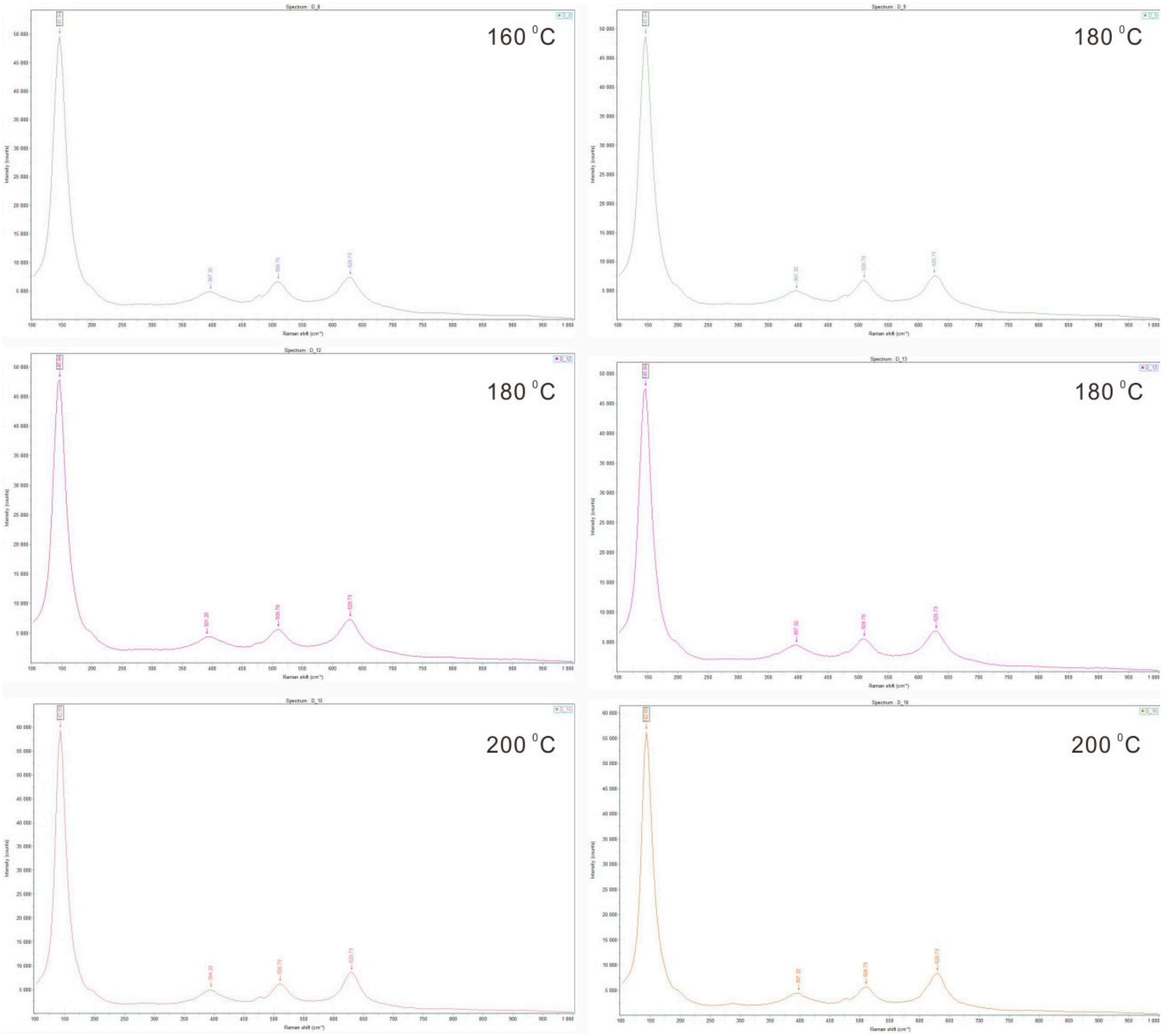

Figure S6. Raman spectra of the anatase TiO_2 microsphere.
